# Retraction: Metagenomic Human Repiratory Air in a Hospital Environment

**DOI:** 10.1371/journal.pone.0147243

**Published:** 2016-01-13

**Authors:** The PLOS ONE Editors

After the publication of the article, readers raised concerns about several aspects of the presentation as well as the methodological aspects of the study and the conclusions drawn. Follow-up with the authors and consultation with editorial board members, identified the following concerns:

- The manuscript was copyedited on submission at the request of the editorial office yet language errors were reintroduced during the evaluation process. The quality of the language of the published article is not in line with the criteria for publication in the journal.
- Fragments of text overlap with text from the "Hospital-acquired infection" entry on Wikipedia: https://en.wikipedia.org/wiki/Hospital-acquired_infection
- Table 1 was reproduced without attribution from: Julia S. Garner: Guidelines for Isolation Precautions in Hospitals Hospital Infection Control Advisory Committee. 01/01/1996, http://wonder.cdc.gov/wonder/prevguid/p0000419/p0000419.asp
- Table 2 was adapted without attribution from: Pasanen, A.L., A review: Fungal exposure assessment in indoor environments. Indoor Air, 2001. 11(2): p. 87–98, http://onlinelibrary.wiley.com/doi/10.1034/j.1600-0668.2001.110203.x/abstract
- 87.7% of sequences in the MG-RAST dataset are noted as failing QC: http://metagenomics.anl.gov/?page=MetagenomeOverview&metagenome=4555914.3
- Evidence that pathogens were detected in the samples is not sufficient.
- Data from MG-RAST have not been validated, either via other bioinformatics methods or independent approaches such as PCR or culture. How the potential non-specificity in the matching to taxons was addressed is not clear.
- For certain genera and species, metagenomics abundance is compared to CFU/mL. No information is provided on how the different species on the agar culture plate were counted or confirmed, or how the normalization steps in library preparation and sequencing were accounted for when measuring metagenomics abundance.
- Whether the results for PhiX174 originate from library preparation or contamination from elsewhere is not reported. The human endogenous retroviruses that are reported are likely reads from the human genome and should be classified as such.
- The accuracy of the taxonomic assignments for the metagenomics reads In Table 7 is unclear. Several of the bacteria listed should not be classified as pathogens.

The Editors consider that the concerns outlined above compromise the integrity of the work and the validity of the conclusions reported and as a result retract this publication.
